# Prenatal diagnosis of Prader–Willi syndrome via maternal UPD15 with placental mosaicism: incidental discovery of fetal DMD carrier status

**DOI:** 10.3389/fgene.2025.1675663

**Published:** 2025-10-30

**Authors:** Yanchou Ye, Yiman Fu, Zhechao Zhang, Haofeng Ning, Fangchao Tao, Xiaonan Wang, Qun Fang, Zheng Chen, Xiulan Hao

**Affiliations:** ^1^ Prenatal Diagnostic Center, Department of Obstetrics and Gynecology, The Seventh Affiliated Hospital, Sun Yat-sen University, Shenzhen, China; ^2^ Center for Reproductive Medicine, The Seventh Affiliated Hospital, Sun Yat-sen University, Shenzhen, China

**Keywords:** T15, UPD15, Prader–Willi syndrome, prenatal diagnosis, confined placental mosaicism, Duchenne muscular dystrophy

## Abstract

**Background:**

Prader–Willi syndrome (PWS) represents a paradigm of genomic imprinting disorders. Given the severe lifelong complications of PWS, prenatal diagnosis is crucial for early intervention and genetic counseling.

**Methods:**

Noninvasive prenatal testing (NIPT) indicated a high risk for fetal trisomy 15 (T15), prompting confirmatory invasive testing. Amniocentesis was performed, and amniotic fluid was analyzed by karyotyping, chromosomal microarray analysis (CMA), trio-based whole-exome sequencing (trio-WES), and short tandem repeat (STR) linkage analysis to investigate the genetic etiology. Post-termination, placental tissue was analyzed by copy number variant sequencing (CNVseq) to evaluate potential mosaicism.

**Results:**

NIPT indicated a suspected T15 (Z-score: 16.4). Subsequent invasive testing confirmed the following: a 13.16 Mb region of homozygosity on chromosome 15q25.1q26.1 and a 273 kb Duchenne muscular dystrophy (DMD) gene deletion on chromosome Xp21.1, both identified by CMA. Trio-WES and STR linkage analysis revealed maternal segmental uniparental disomy of chromosome 15 (UPD15), confirming the genetic basis of PWS. Post-termination, CNVseq further demonstrated confined placental mosaicism (CPM) for T15.

**Conclusion:**

When NIPT suggests a high risk of T15, clinicians should maintain a high suspicion for the “trisomy rescue” mechanism, where an initially trisomic zygote undergoes mitotic correction, ultimately forming UPD15 with CPM. The potential discordance between NIPT and the actual fetal genetic status necessitates definitive prenatal diagnosis, which has critical implications for subsequent pregnancy management. Therefore, the concomitant findings of PWS and DMD carrier status require comprehensive prognostic evaluation and recurrence risk assessment.

## 1 Introduction

PWS is a rare disorder with an estimated incidence of 1/10,000–1/30,000. A recent study screening 16,579 newborns for PWS in Australia found a birth incidence of 1:8,290 (Godler et al., 2022). It is pathologically characterized by the loss of paternal gene expression in the 15q11.2-q13 region. The primary pathogenic mechanisms include gene deletion (65%–75%), maternal UPD (20%–30%), and imprinting defect (1%–3%) (Cassidy et al., 2012; Beygo et al., 2019). The syndrome is clinically defined by a characteristic triad of manifestations: neonatal hypotonia, hyperphagia-induced obesity during infancy, and developmental delay. These core features are frequently accompanied by hypogonadism, cognitive impairment, and distinctive facial characteristics. Patients with deletion are typically the most severely affected, while those with UPD or imprinting defects exhibit a less severe phenotype (Lossie et al., 2001). Differences in body mass index, head circumference, and seizure activity are the most pronounced among the classes (Mahmoud et al., 2021). When PWS coincides with DMD carrier status, additional surveillance for cardiomyopathy and muscular dystrophy is warranted. Prenatal manifestations may include reduced fetal movements and low birth weight, although definitive prenatal diagnosis remains challenging and critically important (Angelis et al., 2015).

The NIPT was based on low-pass genome-wide sequencing. This approach can detect not only autosomal aneuploidies but also copy number variations (CNVs) exceeding 1 Mb in size (Dungan et al., 2023). While NIPT can detect PWS caused by 15q11.2 deletion, it has a high false-positive rate and cannot identify UPD or imprinting defects (Butler, 2017). The definitive diagnosis of PWS requires CMA to detect deletions and STR linkage analysis to distinguish isodisomy and heterodisomy UPD. However, this combination cannot detect imprinting center defects. Trio-WES combined with STR linkage analysis can detect not only UPD but also imprinting center defects. The co-occurrence of PWS and DMD carrier status demands dual-pathway counseling that addresses both the imprinting disorder and the X-linked inheritance pattern.

## 2 Materials and methods

### 2.1 Subjects

This study involved a pregnant woman who received routine prenatal screening at the Prenatal Diagnostic Center of the Seventh Affiliated Hospital of Sun Yat-sen University. NIPT indicated a high-risk result, prompting subsequent invasive prenatal diagnosis. The pregnant woman was 33 years old (G4P3A0), and her current husband was 37 years old. Neither had a significant family genetic history (data collected in 2024). All participants in the present study provided written informed consent. Ethical approval for the study was obtained from the hospital’s ethics committee (No:KY-2025-382-01). Fetal ultrasound was performed at 27 weeks of gestation and estimated that the fetal size was equivalent to 24 weeks gestation. The findings suggested symmetrical intrauterine growth restriction (IUGR) accompanied by decreased fetal movements and oligohydramnios. Placental parenchyma was slightly thicker and more echogenic. Maternal–fetal hemodynamic testing revealed abnormalities: the fetal middle cerebral artery (MCA) peak systolic velocity was elevated (1.35–1.4 MoM). There was significantly increased umbilical artery resistance, resulting in a low cerebroplacental (CP) ratio. These findings were indicative of placental insufficiency. The uterine artery Doppler spectra showed significantly increased resistance bilaterally. Based on these hemodynamic and placental findings, the pregnant woman was assessed to be at a very high risk of eclampsia.

### 2.2 Methods

#### 2.2.1 NIPT

Maternal peripheral blood (5 mL) was collected in Streck Cell-Free DNA BCT® blood collection tubes (Streck, La Vista, NE, United States) at a gestational age of greater than 12 weeks. Cell-free DNA (cfDNA) was extracted from 200 µL of maternal plasma. Library preparation included end repair, adapter ligation, and PCR. Amplified double-stranded DNA was thermally denatured into single-stranded DNA, cyclized, and converted into DNA nanoballs (DNBs). The DNBs were loaded onto sequencing chips and sequenced on the MGISEQ-2000 platform (BGI, Shenzhen, China) at 0.1× average coverage. Raw reads were aligned with genome version GRCh37/hg19 using BWA. Uniquely mapped reads were selected using SAM tags, and PCR duplicates were removed. Z-scores were calculated to detect fetal chromosomal aneuploidies and CNVs.

#### 2.2.2 CMA

Approximately 10 mL of amniotic fluid was collected and centrifuged at 3000 rpm for 10 min to obtain the cell pellet. Genomic DNA (gDNA) for CMA, trio-WES, and STR linkage analysis was then extracted from the pellet using the QIAGEN DNA Mini Kit (Cat. 51306; QIAGEN, Hilden, Germany) per the manufacturer’s instructions. The gDNA concentration was 61.8 ng/μL; 5 µL (309 ng) was used for the CytoScan 750K array (Thermo Fisher Scientific, Waltham, MA, USA), exceeding the optimal 250 ng input. In clinical scenarios with suspected maternal blood contamination, short-term amniocyte culture can be employed to enrich adherent fetal epithelial-like cells and deplete non-adherent maternal lymphocytes; culture was not required in this case due to adequate yield and quality from uncultured cells. Extracted gDNA was amplified, labeled, and hybridized to the Affymetrix CytoScan 750K array following the manufacturer’s protocol. Raw data were analyzed in the Chromosome Analysis Suite (ChAS) and annotated to GRCh37/hg19. CNV calls required ≥50 contiguous probes and a minimum size of ≥100 kb. Runs of homozygosity (ROH) were reported and interpreted in clinical context; large ROH (>10 Mb), particularly when restricted to imprinting chromosomes (Angelis et al., 2015; Dungan et al., 2023; McKenna et al., 2010; Zhang et al., 2020; Hu et al., 2022; Benn and Grati, 2018), prompted evaluation for possible UPD. Detected CNVs were interpreted with reference to the scientific literature and public databases.

#### 2.2.3 Whole-exome sequencing (WES)

Trio-WES was performed on gDNA from the amniotic fluid pellet (which was shared with CMA) and on parental blood gDNA. Libraries were prepared, captured by hybridization (Roche NimbleGen), and sequenced as paired-end 100-bp reads on MGISEQ-2000. Reads were processed with SOAPnuke (Chen et al., 2018) and aligned to GRCh37/hg19 using BWA (Li and Durbin, 2009); GATK was used for SNV/indel calling (McKenna et al., 2010), followed by annotation and filtering against population and disease databases. Variant interpretation followed ACMG/AMP guidelines (Richards et al., 2015) with trio-based segregation, and exome-derived SNP/ROH information was leveraged to assess segmental UPD.

#### 2.2.4 STR linkage analysis

UPD testing was performed on gDNA from the amniotic fluid pellet (which was shared with CMA) and on parental blood gDNA using polymorphic STR markers within the imprinted 15q11–q13 region (Beygo et al., 2019). Multiplex fluorescent PCR was followed by capillary electrophoresis on an ABI 3500 Dx Genetic Analyzer (Thermo Fisher Scientific, United States), and electropherograms were analyzed in GeneMapper v6.0. UPD was inferred by trio segregation across informative loci: isodisomy was defined as a single maternal allele at informative loci, and heterodisomy as non-Mendelian inheritance of two maternal alleles with the absence of paternal contribution; mixed patterns were interpreted as segmental UPD. Quality control included standard allele-calling thresholds (minimum peak height and stutter filters) according to laboratory validation and manufacturer recommendations.

#### 2.2.5 CNVseq

CNVseq was performed on gDNA from the umbilical cord and placental tissues (separately sampled from the fetal/chorionic plate and maternal/basal plate) using the MagPure Buffy Coat DNA Midi KF Kit following the manufacturer’s instructions. Low-pass whole-genome libraries were prepared and sequenced on the MGISEQ-2000 at approximately 0.5× coverage. Reads were aligned to GRCh37/hg19 using BWA, and read-depth-based copy-number profiles were generated. Mosaic trisomy 15 was assessed from normalized copy-number ratios across chromosome 15 and summarized as a percent mosaic fraction for each biopsy and pooled sample. CNVs were interpreted in clinical context with reference to the scientific literature and public databases; analyses met internal QC criteria for mapping performance and coverage uniformity.

## 3 Results

### 3.1 NIPT

**FIGURE 1:**
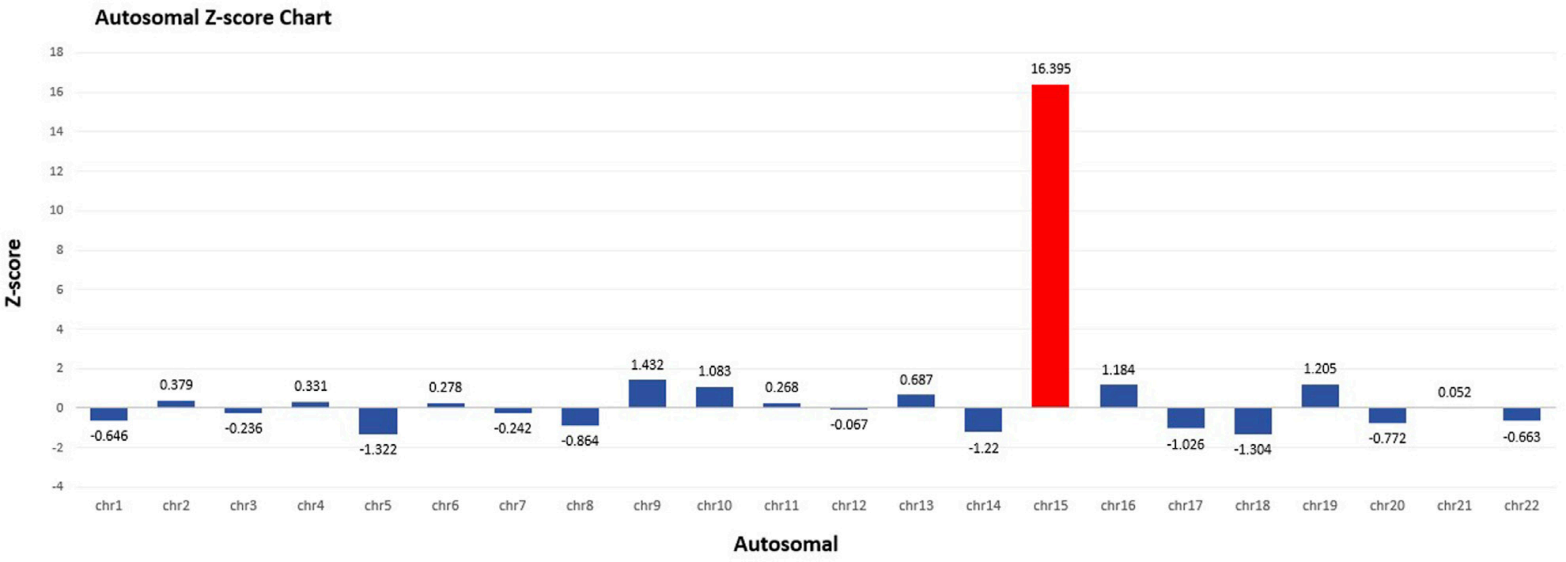
Autosomal Z-score chart in NIPT. Fetal fraction (FF): 11.38%. Z-score for T15 was 16.395.

At 16 weeks of gestation, the fetal NIPT result indicated a high risk for T15. Several quality control metrics were used to evaluate the sequencing data. The fetal DNA fraction was 11.38%. The Q30 score was 95.18%, indicating the proportion of bases with a quality score exceeding 90%. The number of unique reads was 6.63 million, and the Z-score for T15 was 16.395 (Figure 1).

### 3.2 CMA

**FIGURE 2:**
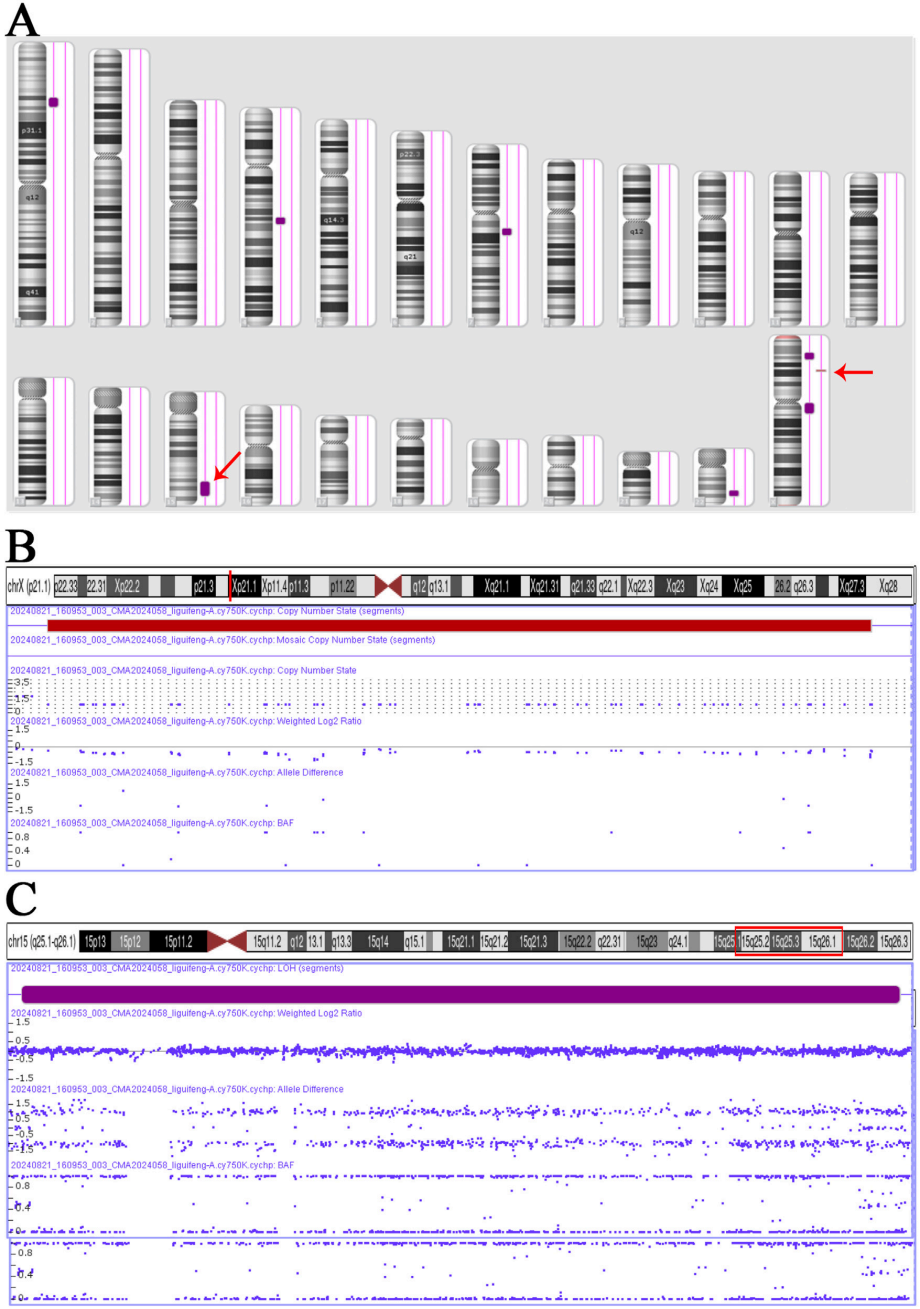
CNV and ROH results in Affymetrix CytoScan 750K CMA. **(A)** Chromosome results. **(B)** Hemizygote deletion pathogenic CNV on Xp21.1, range 31,784,724–32,057,482, fragment length 272.8 kb. **(C)** ROH on 15q25.1q26.1, range 81,006,170–94,168,517, fragment length 13.2 Mb.

The Affymetrix CytoScan 750K CMA identified a heterozygous pathogenic deletion CNV on arr [GRCH37] Xp21.1 (g.31784724_32057482, 272.8 kb) and a region of homozygosity (ROH) on arr [GRCH37] 15q25.1q26.1 (g.81006170_94168517, 13.2 Mb) (Figure 2).

### 3.3 Trio-WES

**FIGURE 3:**
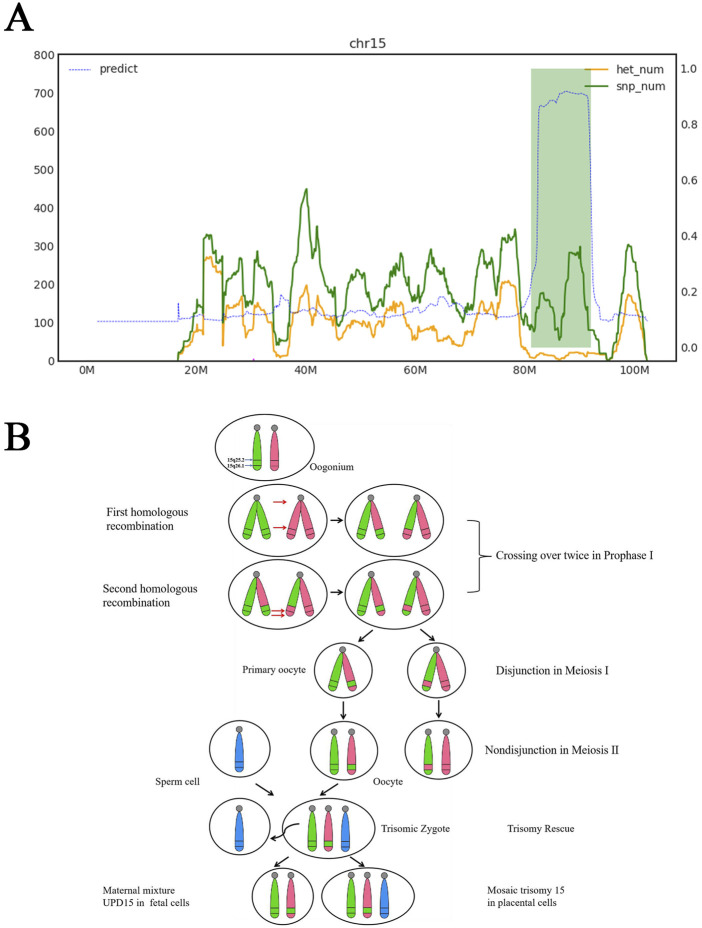
Trio-WES results and the mechanism of segmental maternal UPD15. **(A)** Horizontal axis represents genomic coordinates on chromosome 15. Green curve indicates that single-nucleotide polymorphism (SNP) counts at corresponding positions. Yellow curve displays heterozygous SNP counts at corresponding positions. Left vertical axis denotes gene quantities for green and yellow curves. Right vertical axis is the blue curve, which was the result of calculating ROH, where values approaching 1 indicate regions statistically identified as ROH based on pedigree SNP analysis. Chromosome 15 segment 15q11.1-q25.1 was maternal hetUPD. Chromosome 15 segment 15q25.1-q26.1 was maternal isoUPD, range 81100000–92000000, fragment length 10.9 Mb. Chromosome 15 segment 15q26.1-q26.3 was maternal hetUPD. **(B)** Partial isoUPD was caused by nondisjunction in meiosis Ⅰ after two crossings over, resulting in sections of isodisomy and heterodisomy on the UPD chromosome 15. IsoUPD: isodisomy uniparental disomy, hetUPD: heterodisomy uniparental disomy.

Trio-WES strongly suggests the presence of maternal UPD15, encompassing regions of both isodisomy and heterodisomy. The isodisomy region contained a ∼10.29-Mb ROH spanning 15q25.2q26.1 (GRCH37: g.83328542_93616975). Two heterodisomy regions were identified at 15q11q25.1 and 15q26.1q26.3 (Figure 3A). These findings were consistent with four breaks and two crossover events on chromosome 15 during maternal meiosis Ⅰ (Figure 3B). Additionally, a heterozygous deletion encompassing exons 45–51 of DMD (NM_004006.2: g.31792078_31986631, 194.6 kb) was classified as pathogenic based on the criteria of the ACMG. Maternal testing confirmed that the mother is a carrier of this deletion. Furthermore, this deletion was independently validated by quantitative fluorescent PCR (QF-PCR) (Supplementary Figure S1). No pathogenic or likely pathogenic SNVs were identified.

### 3.4 STR linkage analysis

**FIGURE 4:**
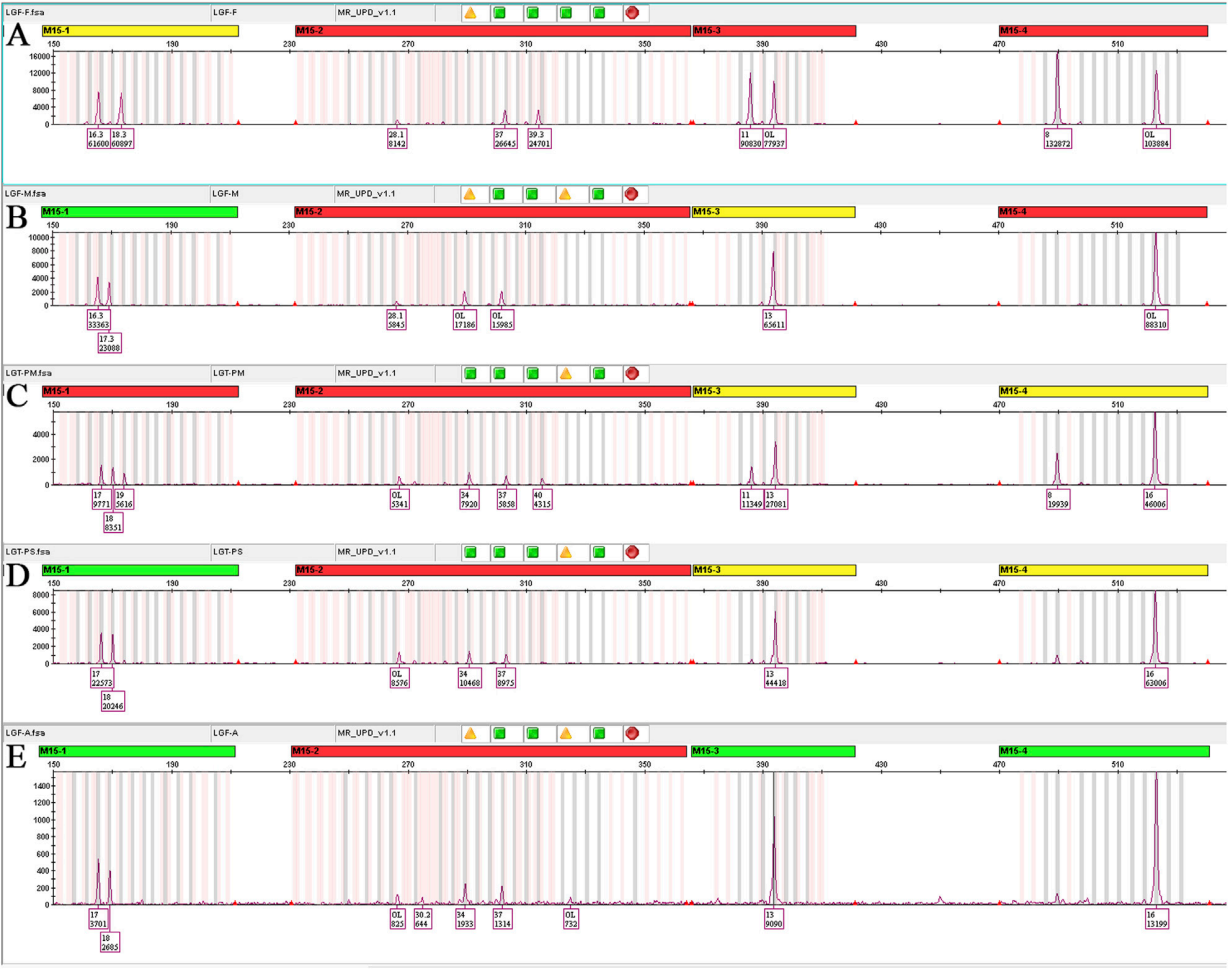
STR linkage analysis results. **(A)** Paternal STR analysis, M15-1 (17,19), M15-2 (37,40), M15-3 (11,13), M15-4 (8,16). **(B)** Maternal STR analysis, M15-1 (17,18), M15-2 (34,37), M15-3 (13), M15-4 (16). **(C)** Placenta ABCDE mix STR analysis, M15-1 (17,18,19), M15-2 (34,37,40), M15-3 (11,13), M15-4 (8,16). These confirmed mosaic T15 as of maternal origin in the maternal surface of the placenta. **(D)** Placenta abcde mix STR analysis, M15-1 (17,18), M15-2 (34,37), M15-3 (13), M15-4 (16); all four STR loci genotypes were fully consistent with maternal origin, with one locus exhibiting biallelic signals. These confirmed maternal hetUPD in the fetal surface of the placenta. **(E)** Amniotic fluid STR analysis, M15-1 (17,18), M15-2 (34,37), M15-3 (13), M15-4 (16); all four STR loci genotypes were fully consistent with maternal origin, with one locus exhibiting biallelic signals. These confirmed maternal hetUPD in the fetus.

STR genotyping of amniotic fluid demonstrated concordance with maternal alleles at all informative 15q11–q13 markers, including one locus with biallelic maternal signals, and no paternal contribution across tested loci. In conjunction with CMA and trio-WES, these segregation patterns confirm maternal segmental UPD15 comprising regions of isodisomy and heterodisomy. Placental STR analysis corroborated the maternal origin of the T15 mosaicism (Figure 4).

### 3.5 CNVseq

CNVseq of umbilical cord tissue identified the heterozygous Xp21.1 deletion and no detectable T15 mosaicism. In contrast, placental sampling demonstrated CPM for T15 with a regional gradient—higher mosaic fraction on the basal (maternal) surface and lower levels on the chorionic (fetal) surface. The Xp21.1 deletion was consistently observed across all tissues. This basal–chorionic gradient accounts for the positive NIPT call and, together with the ultrasound/Doppler evidence of placental insufficiency, supports CPM of likely meiotic origin with post-zygotic trisomy rescue (Supplementary Figure S2; Supplementary Table S1).

## 4 Discussion

Genome-wide NIPT can flag rare autosomal trisomies (A meta-analysis) but shows variable positive predictive value, in part due to fetus–placenta discordance and CPM; therefore, invasive confirmation is recommended when RAT is detected (Zhang et al., 2020; Hu et al., 2022; Taglauer et al., 2014; Konya et al., 2024). On imprinting chromosomes such as 15, trisomy rescue may result in fetal UPD even when the karyotype appears normal (Liehr, 2022; Okuda et al., 2022; Benn and Grati, 2018). Indeed, cases with normal amniotic karyotypes have subsequently been confirmed as maternal UPD15 by methylation/targeted assays, underscoring the need to evaluate UPD despite a normal karyotype (Hong et al., 2023). Although precise estimates vary by chromosome and ascertainment, population-scale studies suggest that UPD arising from trisomy rescue is rare in live-borns (on the order of ∼1 in 2,000–3,500), reinforcing the clinical salience of such presentations (Nakka et al., 2019).

In this study, diagnoses were systematically validated through amniotic fluid analysis combined with a multimodal diagnostic approach encompassing karyotyping, CMA, prenatal ultrasonography, and trio-WES. UPD15 is associated with PWS/AS syndrome, which represents the most classic example of genomic imprinting disorders in humans. In our case, a ROH was initially detected by CMA, which can suggest uniparental isodisomy. However, as the threshold of ROH detection is typically set at 3–10 Mb, smaller regions may be overlooked (Xu et al., 2024). Moreover, ROH may also originate from identity by descent (IBD) rather than from UPD. Furthermore, CMA cannot directly detect uniparental heterodisomy. Trio-WES can interrogate both SNVs/indels and UPD mechanisms (Dharmadhikari et al., 2019). Subsequent Trio-WES revealed segmental UPD15 in the fetus. Additionally, pathogenic variant analysis in the homozygous regions identified no disease-causing mutations. STR linkage analysis further confirmed that both copies of chromosome 15 originated from the pregnant woman. It was hypothesized that this segmental UPD15 resulted from a meiotic nondisjunction event in meiosis Ⅰ followed by two crossover events, leading to a segmental UPD with isodisomy and heterodisomy. Post-fertilization, the zygote exhibited trisomy 15, with trisomy rescue as the likely mechanism by which the aneuploid zygote reverted to euploidy. Placental testing revealed trisomy 15 in the placenta, indicating that during the trisomy rescue process, only the fetal chromosomes were successfully corrected to euploidy, forming CPM (Figure 3B). To validate placental mosaicism, we collected five tissue samples each from the maternal and fetal surfaces of the placenta for CNVseq. The results revealed varying levels of T15 mosaicism across placental regions, with a significantly higher mosaic ratio observed on the maternal than on the fetal surface. This placental mosaicism likely explains why the NIPT indicated T15.

In early pregnancy, if a trisomy 15 rescue event occurs, miscarriage may be averted. Placental abnormalities associated with trisomy 15 may manifest as placental enlargement, structural disorganization, or focal cystic changes. Placental ischemia and hypoxia in such cases could contribute to maternal hypertension and proteinuria, potentially triggering the onset of preeclampsia. Furthermore, insufficient placental perfusion may impair nutrient and oxygen delivery to the fetus, leading to FGR (Eggenhuizen et al., 2021).

In addition to genetic mechanisms, the prenatal clinical features of PWS require special consideration. More than 95% of Prader–Willi syndrome (PWS) cases occur sporadically and lack characteristic fetal structural anomalies (Muthusamy et al., 2020). Gross et al. (2015) conducted interviews with mothers of 106 individuals with PWS and reviewed obstetric records for 47 cases under 10 years of age. They compared prenatal data from PWS pregnancies with those of sibling controls and the general population. The study revealed significantly higher rates of the following in PWS pregnancies compared to controls (p < 0.0001): reduced fetal movements (88%), small for gestational age (SGA) (65%), asymmetrical intrauterine growth (elevated head-to-abdominal circumference ratio, 43%), and polyhydramnios (34%). No major congenital malformations were observed in any PWS cases. Notably, 27%, 29%, and 24% of cases exhibited combinations of two, three, and four of these abnormalities, respectively.

Therefore, when NIPT indicates a high risk for T15 and concurrent ultrasound findings reveal reduced fetal movements and IUGR, raising clinical suspicion for possible PWS due to maternal UPD15, prompt genetic diagnostic testing is strongly recommended. A total of 90 protein coding genes is located within the ROH, spanning approximately 10.3 Mb on chromosome 15. There was an anticipated risk of having a second genetic condition besides PWS (Muthusamy et al., 2020). Trio-WES revealed no pathogenic or likely pathogenic variants within the ROH on 15q25.2q26.1. However, both Trio-WES and CMA consistently identified a hemizygous deletion spanning exons 45–51 of the DMD gene, which was confirmed to be maternally inherited. This discovery underscored that multi-technique integration can detect complex genetic comorbidities, enabling the identification of PWS arising from UPD15 alongside the DMD deletion as a distinct genetic alteration. PWS primarily arises from the loss of paternally expressed genes in the 15q11-q13 region, while DMD is an X-linked recessive disorder that predominantly affects men, with women typically serving as asymptomatic carriers (Kumar et al., 2020). Given the carrier frequency of DMD (1/4,000) (Mo and ser, 1984) and the incidence of PWS (1/8,290), the theoretical co-occurrence probability is 3.02 × 10^−8^. This rare comorbidity underscores the necessity for multidisciplinary collaboration among the disciplines of neurology, endocrinology, and genetics, necessitating comprehensive genetic counseling to address overlapping phenotypes and optimize therapeutic strategies. In this case, the fetus was of female gender with PWS, so she was expected to be a carrier rather than clinically affected by DMD. However, had the fetus been of male gender with PWS, the characteristic neonatal hypotonia of PWS could mask early neuromuscular manifestations of DMD, while progressive muscle weakness caused by the DMD deletion would exacerbate motor dysfunction during infancy, potentially delaying the recognition of both conditions (Brogna et al., 2019). We agree that a maternal X-linked deletion of ∼200–300 kb can be detectable by low-coverage WGS (lc-WGS) NIPT under appropriate binning, GC correction, segmentation, and quality control, as shown by reports of maternal incidental CNVs and maternal DMD CNVs identified on lc-WGS NIPT (Brison et al., 2019; Wang et al., 2024; Brison et al., 2017). In our setting, however, the assay validation and the laboratory reporting policy did not include routine analysis or disclosure of incidental maternal single-gene CNVs at this size range. Specifically, reporting was limited to a predefined set of maternal or maternofetal CNVs linked to specified conditions (Supplementary Table S2); otherwise, maternal incidental CNVs were disclosed only when they exceeded 5 Mb (Cox et al., 2025). Thus, the maternal DMD deletion of exons 45–51 was not analyzed/reported because of validation and policy scope, rather than technical impossibility. For context, haplotype-based single-gene NIPT represents a distinct workflow primarily intended for fetal interrogation of X-linked monogenic disease and is not required for the initial recognition of maternal carrier status on lc-WGS NIPT when such analysis is validated and reportable. In addition, the X-chromosomal cfDNA signal is predominantly maternal-dominated—particularly with a female fetus—limiting reliable fetal inference even when a maternal CNV is detectable; moreover, many laboratories do not routinely analyze or report maternal incidental CNVs in lc-WGS NIPT (Tang et al., 2022). From a long-term management standpoint, women with PWS who are also DMD carriers should receive cardiomyopathy surveillance in accordance with carrier care recommendations (e.g., baseline and periodic assessment with echocardiography and/or cardiac MRI) alongside standard PWS management and reproductive counseling (Birnkrant et al., 2018; Feingold et al., 2017). PWS in this case occurred sporadically, which confers a recurrence risk of less than 1% for future siblings. The mother was confirmed to be a carrier of the maternally inherited pathogenic deletion in the DMD gene, there is a 50% probability for each male offspring to inherit the deletion, and a 50% probability for each female offspring to be a carrier. Given these risks, combined with the technical limitations of routine NIPT, which cannot reliably detect fetal DMD deletion due to insufficient sequencing depth, direct prenatal diagnosis through invasive procedures such as amniocentesis or chorionic villus sampling is strongly recommended in future pregnancies. Pre-implantation genetic testing (PGT) may be considered as an alternative strategy to reduce the transmission risk of both disorders.

## 5 Conclusion

Our study demonstrates how trisomy rescue can result in UPD15 with CPM, underscoring the necessity for invasive diagnostic confirmation when NIPT indicates T15. An integrated multimodal approach utilizing karyotyping, CMA, trio-WES, STR analysis, and CNVseq successfully resolved the discordant findings and identified both PWS and the DMD carrier status. These findings emphasize the critical importance of comprehensive genetic testing in guiding accurate diagnosis and genetic counseling. Clinically, this dual diagnosis requires individualized risk assessment and family-centered management strategies. Despite the inherent limitations of NIPT as a standalone screening tool, our findings strongly support the implementation of integrated genomic diagnostic approaches in contemporary prenatal care.

## Data Availability

The original contributions presented in the study are publicly available. Raw data have been deposited to the National Center for Biotechnology Information (NCBI) under the BioProject with accession number PRJNA1348100, the data can be found here: https://www.ncbi.nlm.nih.gov/bioproject/PRJNA1348100. Further inquiries can be directed to the corresponding author.

## Supplementary material

SUPPLEMENTARY FIGURE S1 QF-PCR results for exons 45 and 51 of the DMD gene. The paternal DMD gene exons 45 and 51 are consistent with normal male control, indicating a normal result. The maternal DMD gene exons 45 and 51 show approximately 50% of the level in normal female control, therefore demonstrating heterozygosity deletion. The fetal DMD gene exons 45 and 51 are consistent with the maternal profile, confirming heterozygosity deletion. NC(F): normal female control, NC(M): normal male control.

SUPPLEMENTARY FIGURE S2 Placenta samples in CNVseq. Ⅰ. Placental marking A, B, C, D, and E refer to tissues on the fetal surface of the placenta. Ⅱ. Placental marking a, b, c, d, and e refer to tissues on the maternal surface of the placenta.

SUPPLEMENTARY TABLE S1 CNVseq results of fetal tissue and placental tissue. Placental side A, B, C, D, and E refer to tissues on the surface of the fetal placenta. Placental side a, b, c, d, and e refer to tissues on the maternal surface of the placenta. The placenta ABCDE mix consists of equal proportions of DNA from the five positions of placenta A, B, C, D, and E. The placenta abcde mix consists of equal proportions of DNA from the five positions of placenta a, b, c, d, and e.

SUPPLEMENTARY TABLE S2 Reporting framework for maternal and maternofetal CNVs in lc-WGS NIPT (laboratory validation and policy scope).
