# Supplementary material for: Prenatal diagnosis of Prader–Willi syndrome via maternal UPD15 with placental mosaicism: incidental discovery of fetal DMD carrier status
Source: Front Genet. 2025 Oct 30;16:1675663. doi: 10.3389/fgene.2025.1675663 (PMC12611562; doi:10.3389/fgene.2025.1675663)
Supplement: Supplementary file 2 [file Table1.docx]

| Number | Sample | CNV | Mosaic T15 |
| --- | --- | --- | --- |
| 1 | umbilical cord | sseq[GRCh37]Xp21.1p21.1(31794125_32060919)×1 | 0 |
| 2 | placenta ABCDE mix | sseq[GRCh37]Xp21.1p21.1(31794125_32060919)×1 | 12% |
| 3 | placenta abcde mix | sseq[GRCh37]Xp21.1p21.1(31794125_32060919)×1 | 74% |
| 4 | placenta side A | sseq[GRCh37]Xp21.1p21.1(31794125_32060919)×1 | 10% |
| 5 | placenta side B | sseq[GRCh37]Xp21.1p21.1(31794125_32060919)×1 | 24% |
| 6 | placenta side C | sseq[GRCh37]Xp21.1p21.1(31794125_32060919)×1 | 11% |
| 7 | placenta side D | sseq[GRCh37]Xp21.1p21.1(31794125_32060919)×1 | 14% |
| 8 | placenta side E | sseq[GRCh37]Xp21.1p21.1(31794125_32060919)×1 | 6% |
| 9 | placenta side a | sseq[GRCh37]Xp21.1p21.1(31794125_32060919)×1 | 64% |
| 10 | placenta side b | sseq[GRCh37]Xp21.1p21.1(31794125_32060919)×1 | 100% |
| 11 | placenta side c | sseq[GRCh37]Xp21.1p21.1(31794125_32060919)×1 | 49% |
| 12 | placenta side d | sseq[GRCh37]Xp21.1p21.1(31794125_32060919)×1 | 74% |
| 13 | placenta side e | sseq[GRCh37]Xp21.1p21.1(31794125_32060919)×1 | 100% |

Table 1. CNV-seq results of fetal tissue and placental tissue

Placental side A, B, C, D and E refer to tissues on the surface of the fetal placenta. Placental side a, b, c, d and e refer to tissues on the maternal surface of the placenta. The placenta ABCDE mix consists of equal proportions of DNA from the five positions of placenta A, B, C, D, and E. The placenta abcde mix consists of equal proportions of DNA from the five positions of placenta a, b, c, d, and e. CNV means copy number variation. T15 means trisomy chromosomal 15.
