# Supplementary figures and images for: Prenatal diagnosis of Prader–Willi syndrome via maternal UPD15 with placental mosaicism: incidental discovery of fetal DMD carrier status
Source: Front Genet. 2025 Oct 30;16:1675663. doi: 10.3389/fgene.2025.1675663 (PMC12611562; doi:10.3389/fgene.2025.1675663)

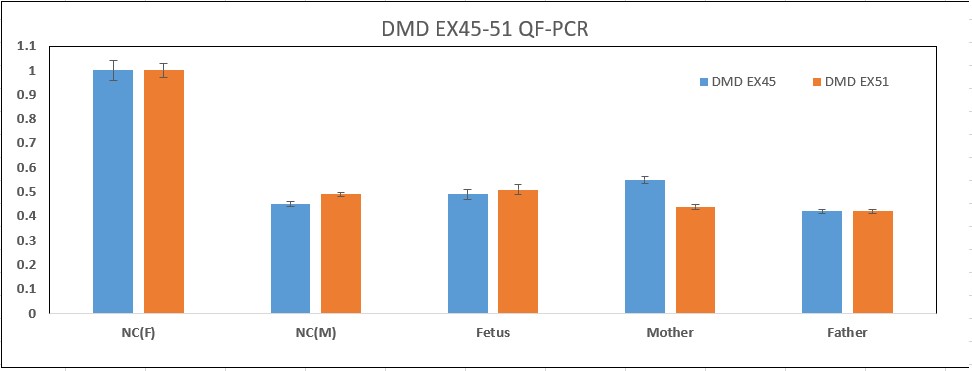

Supplement: Supplementary file 3 [file Image1.jpeg]

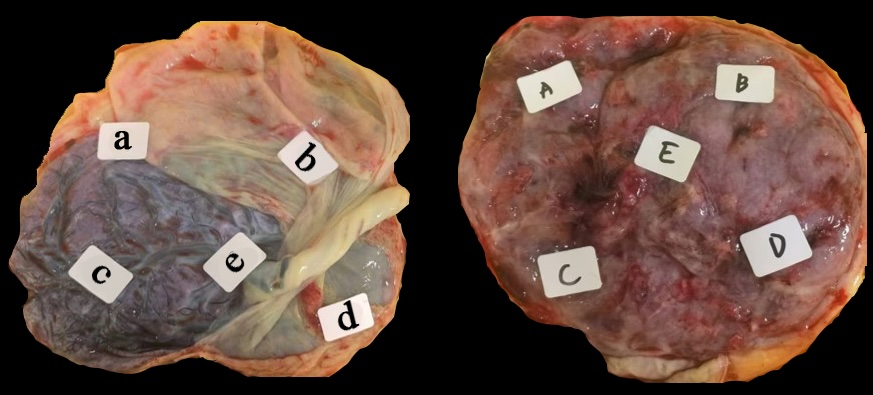

Supplement: Supplementary file 4 [file Image2.jpeg]
